# Comparative analysis of albumin quotient and total CSF protein in immune-mediated neuropathies: a multicenter study on diagnostic implications

**DOI:** 10.3389/fneur.2023.1330484

**Published:** 2024-01-09

**Authors:** Tabea Seeliger, Stefan Gingele, Yunus Emre Güzeloglu, Lena Heitmann, Benjamin Lüling, Felix Kohle, Hannah Preßler, Frauke Stascheit, Jeremias Motte, Anna Lena Fisse, Thomas Grüter, Kalliopi Pitarokoili, Thomas Skripuletz

**Affiliations:** ^1^Department of Neurology, Hannover Medical School, Hannover, Germany; ^2^Department of Neurology, St Josef Hospital, Ruhr University Bochum, Bochum, Germany; ^3^Department of Neurology, Faculty of Medicine and University Hospital of Cologne, University of Cologne, Cologne, Germany; ^4^Department of Neurology with Experimental Neurology, Charité – Universitätsmedizin Berlin, Corporate Member of Freie Universität Berlin and Humboldt Universität, Berlin, Germany; ^5^Neuroscience Clinical Research Center, Charité — Universitätsmedizin Berlin, Corporate Member of Freie Universität Berlin, Humboldt-Universität zu Berlin, Berlin, Germany

**Keywords:** Q_Alb_, blood-cerebrospinal fluid-barrier, CSF, barrier-dysfunction, immune-mediated neuropathies

## Abstract

**Introduction:**

Blood-cerebrospinal fluid (CSF) barrier dysfunction is pivotal for diagnosing immune-mediated neuropathies, especially in spinal nerve root inflammation. Typically, either total CSF protein or the CSF to serum albumin ratio (Q_Alb_) is measured. Total CSF protein measurements have limitations, notably its fixed reference value regardless of age, in contrast to the age-dependent reference for Q_Alb_. Our goal was to evaluate both markers in patients with immune-mediated neuropathies.

**Methods:**

In our multicenter research, we collected retrospective CSF data from patients suffering from immune-mediated neuropathies across four German research centers. These parameters were analyzed in relation to their clinical characteristics.

**Results:**

Out of 419 samples, 36 (8.6%) displayed a notable variation between total CSF protein and Q_Alb_ values. A detailed analysis revealed that patients displaying elevated Q_Alb_ but normal total CSF protein levels were significantly younger at disease onset (*p* = 0.01), at the time of diagnosis (*p* = 0.005), and when undergoing lumbar puncture (*p* = 0.001) compared to patients with elevated CSF protein and normal Q_Alb_ levels. These effects were especially evident for the subgroup of samples derived by female patients.

**Discussion:**

Our work confirms the crucial role of Q_Alb_ in diagnosing immune-mediated neuropathies and particularly its efficacy as a marker for evaluating the blood-CSF barrier in patients with an earlier disease onset. Considering the significance of the albumin quotient, its assessment is especially advisable in younger patients of female sex to avoid missing a potential barrier dysfunction that might be falsely negative when using total protein.

## 1 Introduction

Blood-cerebrospinal fluid (CSF) barrier dysfunction is one of the hallmarks of the local inflammation at the spinal nerve roots that characterizes autoimmune-mediated neuropathies such as Guillain-Barré syndrome (GBS) and chronic inflammatory demyelinating polyneuropathy (CIDP) (1, 2). Information of the blood-CSF barrier function therefore plays a crucial role in the diagnosis and subsequent management of patients with immune-mediated neuropathies. Therefore, CSF analysis is included in the European Academy of Neurology/Peripheral Nerve Society (EAN/ PNS) diagnostic consensus guidelines for CIDP and multifocal motor neuropathy (MMN) (3, 4), as well as in the diagnostic workup of GBS (5).

Albumin, which is solely produced in the liver, is the most abundant protein within the CSF, even though it is not produced or catabolized intrathecally (6). Therefore, the ratio of CSF and serum albumin concentrations (Q_Alb_) has been proposed as the preferred marker of blood-CSF-barrier function (7). But more importantly, the interpretation of Q_Alb_ is facilitated by age-dependent upper reference limits (Q_Alb_ lim) (8–10).

An alternative and still widely implemented approach to assess the blood-CSF-functionality is represented by the total CSF protein level. Although measurements of total protein in CSF are readily available, this traditional parameter has several disadvantages. The absolute CSF protein concentration is dependent on the serum protein concentration, age, CSF flow rate, and assay type (11). Also, a possible intrathecal synthesis of antibodies can influence total CSF protein values (12). Therefore, the measurement of absolute protein concentration in CSF may show normal results even though blood-CSF-barrier dysfunction is present (13).

Since the identification of a blood-CSF-barrier-dysfunction is needed for the diagnosis of inflammatory polyneuropathies and in particular for CIDP, a sensitive detection method is highly relevant in clinical routine. This is especially important considering that normal reference values for both parameters in healthy populations have been well studied (14–16). Therefore, our aim was to compare the applicability of Q_Alb_ and total CSF protein levels in a large number of patients with immune-mediated neuropathies.

## 2 Methods

### 2.1 Patient recruitment

In this multicenter study, CSF parameters of patients with autoimmune-mediated neuropathies were retrospectively recorded at four German study sites {Hannover Medical School (MHH), Ruhr-University Bochum [Department of Neurology, University Hospital St. Josef-Hospital] (UK-RUB), Faculty of Medicine and University Hospital of Cologne, Charité- Universitätsmedizin Berlin}, that participate in the German neuritis network “Neuritis Netz” (17). Study inclusion required that patients over the age of 18 years independent of disease duration and severity met the national and European guidelines for diagnosis of immune-mediated neuropathy according to EAN criteria at time of sampling. Only samples with information on total CSF protein and CSF and serum albumin were considered.

### 2.2 Assessment of CSF/ serum parameters and clinical data

Albumin concentrations in CSF and serum were determined using kinetic nephelometry. At Hannover Medical School, the Beckman Coulter IMMAGE was utilized, at UK-RUB both the Beckman Coulter IMMAGE 800 and Siemens Atellica^®^ NEPH 630 System were used, at the University Hospital of Cologne the BN ProSpec was employed, and in Berlin, turbidimetry with the cobas®8,000-modul 701 was adopted. The CSF total protein levels were measured at MHH using a Beckman Coulter spectrophotometer, at UK-RUB using diasys respons^®^910, and in Berlin and Cologne by turbidimetry with a Cobas system (Cologne: Cobas c702).

The CSF albumin/serum albumin ratio was expressed as Q_Alb_. The age-dependent upper reference limit for Q_Alb_ (Q_Alb_ lim) was calculated according to the established formula suggested by Reiber and Trendelenburg: Q_Alb_ lim = (age in years/15) + 4 (9, 10). Q_Alb_ values higher than Q_Alb_ lim were considered as elevated. CSF total protein values >500 mg/L were reported as elevated. Further CSF parameters included cell count, q-IgG, q-IgA, q-IgM, and evidence of oligoclonal bands. In addition, socio-demographic data including baseline information on diagnosis and symptom onset was collected.

At the time of CSF analysis, disease severity and impairment levels were assessed using two metrics. First, the Inflammatory Neuropathy Cause and Treatment (INCAT) disability score was utilized. This score evaluates both upper and lower limb functionality, with a total score range of 0–10 (18). Additionally, the Medical Research Council (MRC) Sum Score was employed for eight distinct muscle groups. These groups included upper extremity abduction, elbow flexion, wrist extension, index finger abduction, hip flexion, knee extension, foot dorsiflexion, and great toe dorsiflexion. The total score for this metric ranges from 0 to 80 (19, 20).

An effect of the contributing centers or of the diagnoses was ruled out for all subgroup analyses.

### 2.3 Statistical analysis

The data were handled descriptively. Continuous variables were first tested for normal distribution using the Shapiro–Wilk test. Subsequently, parametric variables were described as mean and standard deviation (SD) and non-parametric variables as median and interquartile range (IQR). Subgroup analysis was performed using chi^2^-test or Fisher’s exact test for categorical data as appropriate and Mann–Whitney-U-test for metric variables without normal distribution. Correlation analysis was performed using Spearman’s rank correlation. Furthermore, logistic regression analysis was performed for candidate variable in a univariable manner and for variables with significant effects additionally in a multivariable manner. value of ps <0.05 were considered statistically significant. Statistical analysis was performed by STATA^®^ V16.1 (StataCorp LLC, Texas, United States).

## 3 Results

### 3.1 Patients’ characteristics

**Figure 1:**
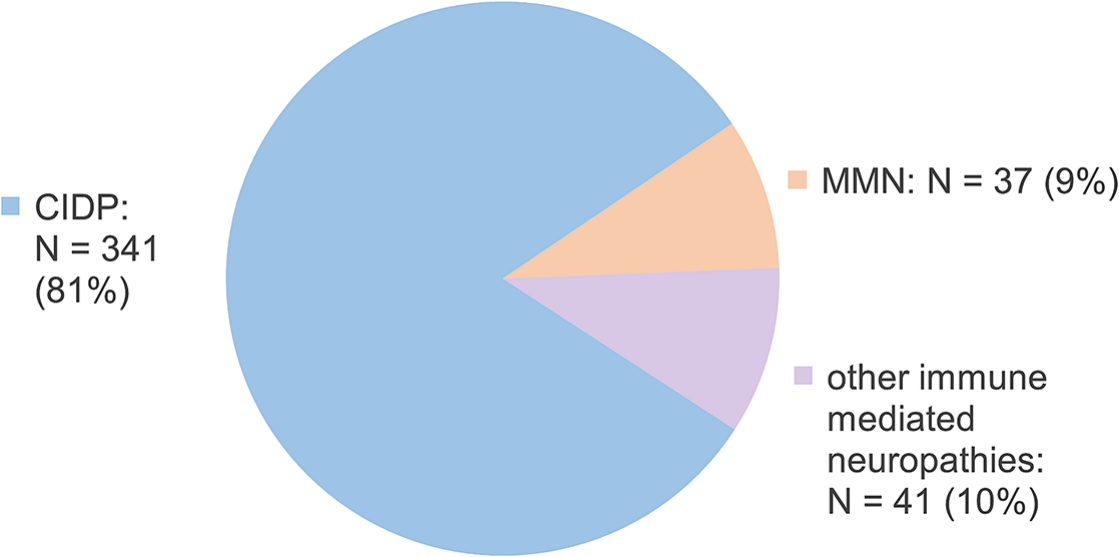
Currently prompted diagnoses for included samples. N, number; CIDP, chronic inflammatory demyelinating polyneuropathy; MMN, multifocal motor neuropathy; GBS, Guillain-Barré syndrome.

Information on 419 CSF samples was assessed at four study sites in Germany. Based on a repeated lumbar puncture in 30 cases, the samples were derived from a total of 389 included patients with a median age of 59 [IQR 51–68] years of which 72% were of male sex (n = 280) suffering from immune-mediated neuropathies. Samples from therapy-naïve patients had been collected between 04/1996 and 11/2022. Baseline data are shown in Table 1 and the current diagnoses for the included samples are shown in Figure 1. CIDP diagnosis was based on the latest diagnostic criteria available at the time-point of sampling.

**Table 1:** Baseline information on included samples and corresponding patients.

	All	Samples derived from male patients	Samples derived from female patients	Value of *p*
Number of samples	419	294 (70%)	125 (30%)	n/a
Number of corresponding patients	389	280 (72%)	109 (28%)	n/a
Age at onset (Median, IQR) [years]	54 (46–63)	55 (47–63)	52 (44–65)	0.2
Age at diagnosis (Median, IQR) [years]	58 (50–68)	59 (51–68)	55 (48–68)	0.07
Age at lumbar puncture (Median, IQR) [years]	59 (50–68)	59 (52–67)	56 (49–70)	0.07
INCAT at time of lumbar puncture (Median, IQR)	3 (1–4)	2 (1–3)	3 (2–4)	**0.007**
MRC Sum Score at time of lumbar puncture (Median, IQR)	76 (70–79)	76 (71–79)	76 (70–79)	0.3
Samples included at Hannover, N (%)	250 (60%)	162 (65%)	88 (35%)	n/a
Samples included at Bochum, N (%)	125 (30%)	97 (78%)	28 (22%)	n/a
Samples included at Köln, N (%)	34 (8%)	26 (76%)	8 (24%)	n/a
Samples included at Berlin, N (%)	10 (2%)	9 (90%)	1 (10%)	n/a
Diagnosis of CIDP, N (%)	341 (81%)	242 (82%)	99 (79%)	n/a
Diagnosis of MMN, N (%)	37 (9%)	21 (7%)	16 (13%)	n/a
Diagnosis of other autoimmune mediated neuropathies, N (%)	41 (10%)	31 (11%)	10 (8%)	n/a

IQR, interquartile range; N, number; INCAT, Inflammatory Neuropathy Cause and Treatment; MRC, Medical Research Council; bold letters indicating statistically significant findings.

### 3.2 Blood-CSF dysfunction markers

**Figure 2:**
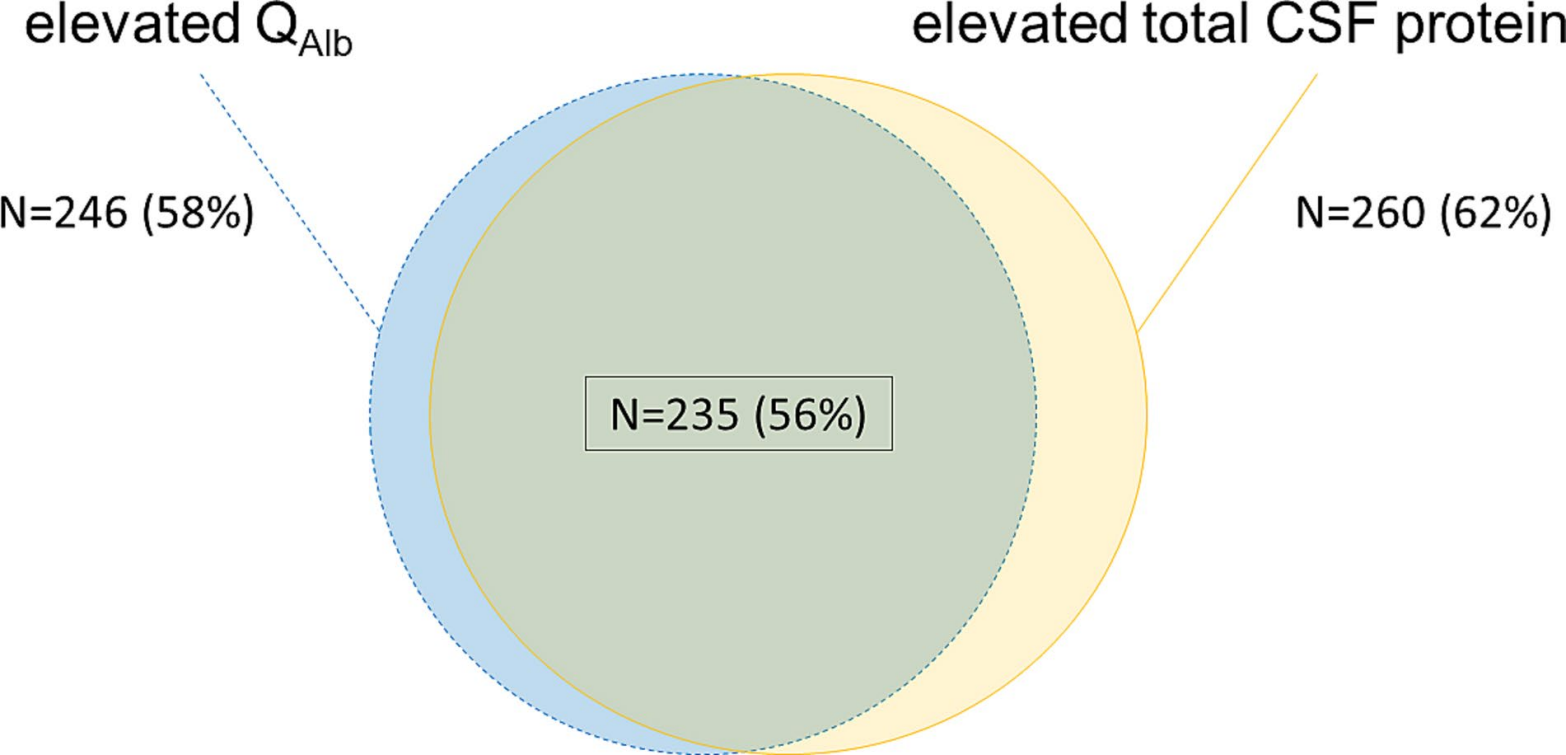
Comparison of the blood-CSF dysfunction-markers Q_Alb_ and total CSF protein. Blue/ dotted lines—elevated Q_Alb_ calculated by age-dependent Reiber-formula (21); yellow—elevated total CSF protein >500 mg/L; green overlapping background/ black frame—both elevated Q_Alb_ and elevated total CSF protein as previously defined. Percentage values refer to the total sample size of 419 samples. N, number; CSF, cerebrospinal fluid; Q_Alb_, ratio of CSF and serum albumin concentrations.

246 samples (58%) showed elevated Q_Alb_ values, while 260 samples (62%) showed elevated total CSF protein values. 25 of 419 samples (6%) had elevated total CSF protein levels without elevated Q_Alb_, while Q_Alb_ was solely elevated in 11 of 419 samples (2.6%). The detailed analysis is depicted in Figure 2.

A first subgroup analysis revealed, that samples with elevated Q_Alb_ and normal total CSF protein values derived from patients of significantly younger age at onset (*p* = 0.01), significantly younger age at diagnosis (*p* = 0.005) and significantly younger age at lumbar puncture (*p* = 0.001) than samples with elevated total CSF protein values and normal Q_Alb_. The different contributing study sites (*p* = 0.3 and *p* = 0.6) or the corresponding diagnoses (*p* = 1.0 and 0.8) did not prove to be significant confounders for this subgroup analysis.

A second subgroup analysis showed the same significant relations for the comparison of samples with isolated elevated Q_Alb_ values versus all samples with elevated total CSF protein values [significantly younger age at onset (*p* = 0.04), diagnosis (*p* = 0.03) and at lumbar puncture (*p* = 0.04)]. In this comparison, INCAT scores at time of lumbar puncture were significantly higher for patients with elevated total CSF protein values independent of Q_Alb_ values compared to patients with isolated elevated Q_Alb_.

Clinical characteristics of all the above-mentioned sample subgroups are shown in Table 2.

**Table 2:** Clinical characteristics of samples with isolated pathological changes for either Q_Alb_ (group A) or total CSF protein (group B) in comparison with all samples, that showed elevated total CSF protein values.

	Elevated Q_Alb_ and normal total CSF protein (group A)	Elevated total CSF protein and normal Q_Alb_ (group B)	Value of *p* (group A vs. group B)	Elevated total CSF protein independent of Q_Alb_ (group C)	Value of *p* (group A vs. group C)
Number of samples	11	25	N/A	260	N/A
Samples derived from male patients, number (%)	9 (82)	17 (68)	0.4	199 (77%)	0.7
Age at onset (Median, IQR) [years]	48 (44–48)	59 (51–59)	**0.01**	54 (46–63)	**0.04**
Age at diagnosis (Median, IQR) [years]	53 (33–57)	63 (56–74)	**0.005**	58 (50–68)	**0.03**
Age at lumbar puncture (Median, IQR) [years]	55 (35–57)	68 (58–73)	**0.001**	59 (51–68)	**0.04**
INCAT at time of lumbar puncture (Median, IQR)	1 (1–2)	2 (1–3)	0.2	3 (1–4)	**0.02**
MRC Sum Score at time of lumbar puncture (Median, IQR)	78 (75–80)	76 (70–79)	0.06	76 (70–79)	0.08
Center effect	n/a	n/a	0.3	n/a	0.6
Effect of diagnosis	n/a	n/a	1.0	n/a	0.8

CSF, cerebrospinal fluid; IQR, interquartile range; INCAT, Inflammatory Neuropathy Cause and Treatment; MRC, Medical Research Council; Q_Alb_, ratio of CSF and serum albumin concentration; bold letters indicating statistically significant findings.

Even though sex distribution did not differ significantly between the previously described subgroups, there have been many studies implying a sexual bias with significantly higher Q_Alb_ and total CSF protein values in male compared to female patients (22). We therefore reassessed both previously conducted subgroup analyses for male and female patients separately: This showed concordant findings for age at diagnosis and at lumbar puncture for male and female patients in the comparison of samples with elevated Q_Alb_ and normal total CSF protein values versus samples with elevated total CSF protein values and normal Q_Alb_ (significantly younger age at diagnosis (males *p* = 0.03, females *p* = 0.04) and at lumbar puncture (males *p* = 0.02, females *p* = 0.04). However, the same age dependent effect was only present in samples derived from female patients when comparing samples with isolated elevated Q_Alb_ values versus all samples with elevated total CSF protein values [significantly younger age at diagnosis (males *p* = 0.1, females *p* = 0.03) and at lumbar puncture (males *p* = 0.1, females *p* = 0.03)]. The full workup is included in Table 3.

**Table 3:** Influence of sex on clinical characteristics of samples with isolated pathological changes for either Q_Alb_ (group A) or total CSF protein (group B) in comparison with samples, that showed elevated total CSF protein values.

Subanalysis of male patients					
	Elevated Q_Alb_ and normal total CSF protein (group A)	Elevated total CSF protein and normal Q_Alb_ (group B)	Value of *p* (group A vs. group B)	Elevated total CSF protein independent of Q_Alb_ (group C)	Value of *p* (group A vs. group C)
Number of samples	9/11 (82%)	17/25 (68%)	0.4	199/260 (77%)	0.7
Age at onset (Median, IQR) [years]	51 (40–57)	59 (53–70)	0.05	54 (46–62)	0.2
Age at diagnosis (Median, IQR) [years]	55 (53–57)	63 (56–74)	**0.03**	59 (51–66)	0.1
Age at lumbar puncture (Median, IQR) [years]	55 (54–57)	70 (57–73)	**0.02**	59 (52–66)	0.1
INCAT at time of lumbar puncture (Median, IQR)	1 (1–2)	2 (1–3)	0.3	2 (1–4)	**0.03**
MRC Sum Score at time of lumbar puncture (Median, IQR)	78 (78–80)	78 (71–78)	0.2	76 (70–79)	0.09
Center effect	n/a	n/a	0.7	n/a	0.3
Effect of diagnosis	n/a	n/a	1.0	n/a	0.4
subanalysis of male patients					
Number of samples	2/11 (18%)	8/25 (32%)	0.4	61/260 (23%)	0.7
Age at onset (Median, IQR) [years]	30 (28–32)	62 (43–71)	**0.0495**	52 (44–69)	**0.04**
Age at diagnosis (Median, IQR) [years]	31 (29–33)	64 (55–72)	**0.04**	54 (48–70)	**0.03**
Age at lumbar puncture (Median, IQR) [years]	31 (30–32)	66 (59–72)	**0.04**	55 (48–70)	**0.03**
INCAT at time of lumbar puncture (Median, IQR)	2 (1–3)	4 (2–4)	0.3	3 (2–5)	0.3
MRC Sum Score at time of lumbar puncture (Median, IQR)	76 (74–78)	72 (67–76)	0.2	74 (60–78)	0.6
Center effect	n/a	n/a	0.4	n/a	0.6
Effect of diagnosis	n/a	n/a	1.0	n/a	1.0

CSF, cerebrospinal fluid; IQR, interquartile range; INCAT, Inflammatory Neuropathy Cause and Treatment; MRC, Medical Research Council; Q_Alb_, ratio of CSF and serum albumin concentrations; bold letters indicating statistically significant findings.

We further conducted a logistic regression analysis for all samples with elevated Q_Alb_ and for all samples with elevated total CSF protein values for better understanding of sex-, age- and disability-dependent effects on both markers: Therein, samples from female patients showed significantly less often elevated Q_Alb_ (odds ratio [OR] 0.39 [95% confidence interval [CI] 0.26–0.61], *p* < 0.001) and total CSF protein values (0.46 [95% CI 0.3–0.7], *p* < 0.001). Also, age at lumbar puncture was significantly younger for samples with elevated Q_Alb_ (OR 0.98 [95% CI 0.97–0.996], *p* = 0.04). The full logistic regression analysis is detailed in Supplementary Table 1.

### 3.3 Overall interrelation between Q_Alb_ and total CSF protein

**Figure 3:**
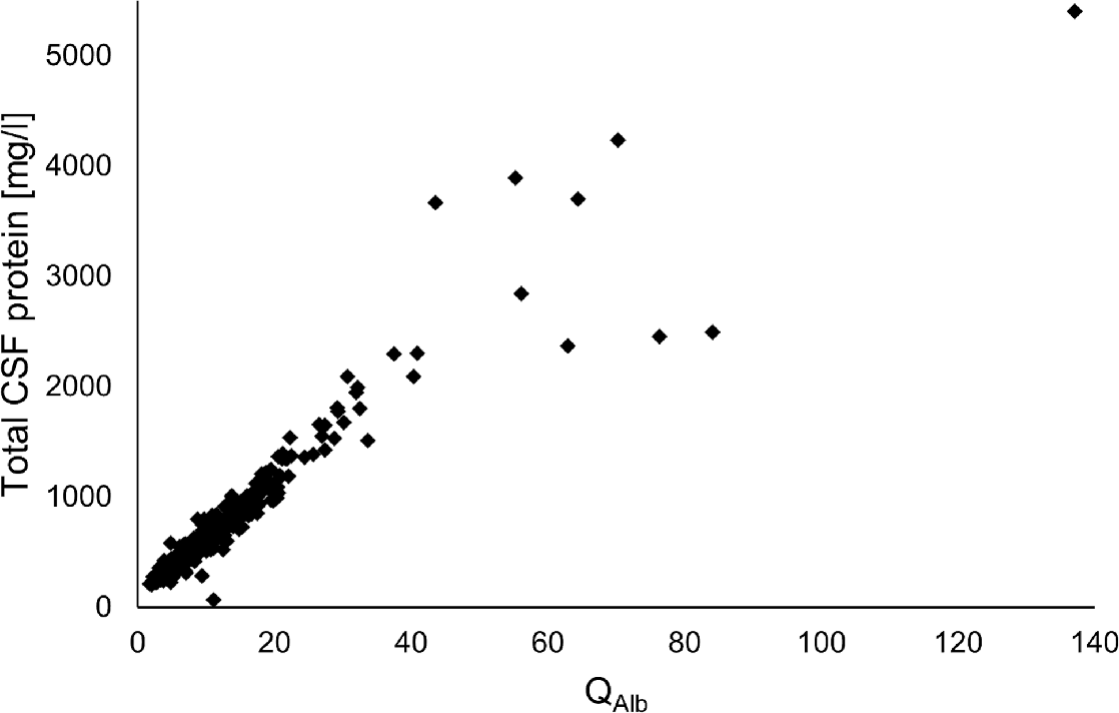
Correlation analysis of Q_Alb_ and total CSF protein values (Spearman’s rho coefficient = 0.96, *p* < 0.0001). CSF, cerebrospinal fluid; Q_Alb_, ratio of CSF and serum albumin concentrations.

Q_Alb_ and total CSF protein values showed a highly positive correlation (Spearman’s rho coefficient = 0.96, *p* < 0.0001). The corresponding data are graphically presented in Figure 3.

Concerning the interrelation between blood-CSF dysfunction and disease severity scores (depicted in Supplementary Figure 1), a marginal inverse correlation was found for Q_Alb_ and the MRC Sum score at the time of lumbar puncture (Spearman’s rho coefficient = −0.098, *p* = 0.048). Further correlation analysis did not reveal a statistical relationship for total CSF protein values with the respective MRC Sum (Spearman’s rho coefficient = −0.08, *p* = 0.1), or INCAT scores (Spearman’s rho coefficient = 0.06, *p* = 0.2), as well as for Q_Alb_ and the respective INCAT scores (Spearman’s rho coefficient = 0.09, *p* = 0.08).

The full workup of the remaining CSF parameters is provided in Table 4.

**Table 4:** Detailed overview of the CSF parameters.

Included CSF parameters	Information available, N (male/female)	Absolute values, all	Absolute values, males	Absolute values, females	Value of *p*
Cell count, median (IQR) [per μl]	320 (212/108)	1.3 (0.7–2.7)	1.3 (0.7–2.7)	1.3 (0.9–2.3)	0.8
Total CSF protein, median (IQR) [mg/l]	419 (294/125)	570 (425–800)	607 (457–832)	477 (372–652)	**<0.0001**
Q_Alb_, median (IQR)	419 (294/125)	8.9 (6.3–13.3)	9.5 (7–14.2)	7.2 (5.1–10.4)	**<0.0001**
q-IgG, median (IQR)	416 (292/124)	4.4 (3–6.7)	4.8 (3.3–7.4)	3.6 (2.4–5.5)	**<0.0001**
q-IgA, median (IQR)	402 (282/120)	2.4 (1.6–3.9)	2.6 (1.7–4.3)	2 (1.3–3.1)	**<0.0001**
q-IgM, median (IQR)	383 (269/114)	0.9 (0.5–1.8)	1 (0.6–1.8)	0.8 (0.4–1.6)	0.009
Intrathecal IgG synthesis, median (IQR) [%]	365 (254/111)	0 (0–0)	0 (0–0)	0 (0–0)	0.2
Intrathecal IgA synthesis, median (IQR) [%]	358 (249/109)	0 (0–0)	0 (0–0)	0 (0–0)	0.3
Intrathecal IgM synthesis, median (IQR) [%]	342 (240/102)	0 (0–0)	0 (0–0)	0 (0–0)	0.9
Oligoclonal bands, N (%)	415 (293/122)	24 (6%)	19 (6.5%)	5 (4%)	0.3
Lactate, median (IQR) [mm/l]	409 (286/123)	1.7 (1.5–1.9)	1.7 (1.5–1.9)	1.6 (1.5–1.8)	0.2

CSF, cerebrospinal fluid; IQR, interquartile range; Q_Alb_, ratio of CSF and serum albumin concentrations; N, number.

## 4 Discussion

In clinical settings, total CSF protein levels remain a popular choice for evaluating the blood-CSF-barrier function. While this method might seem advantageous owing to its straightforwardness and cost-efficiency (only needing one CSF sample instead of both CSF and serum samples for the albumin quotient), it is not without its challenges. A host of confounding factors can complicate the interpretation, making the procedure less reliable than it may initially appear (7).

Evaluating solely the total CSF protein levels without accounting for serum protein concentrations is troubled with inaccuracies. A bulk of CSF proteins have their origin in the plasma. Therefore, when the CSF protein level is interpreted in isolation, without considering the serum protein level, it becomes a source of potential error. Factors such as liver and renal diseases can cause hypoproteinemia or paraproteinemia, and treatments like intravenous immunoglobulins can cause hyperproteinemia, subsequently changing the true total CSF protein levels. This means the straightforward measure of absolute protein concentrations in the CSF could misrepresent the actual situation, potentially indicating a normal scenario even in the face of blood-CSF barrier dysfunction or vice versa.

For example, conditions causing kidney damage, like hemolytic-uremic syndrome, can result in a significant loss of plasma proteins via the urine. Studies have shown that when relying solely on total CSF protein measurements, blood-CSF-barrier dysfunction might go unnoticed due to reduced serum albumin levels (13). However, when adjusting for the serum albumin concentration by determining the Q_Alb_, the barrier dysfunction became evident. In certain cases, patients with renal issues showed only a mild elevation in total CSF protein content, while a much more severe barrier dysfunction was evident upon evaluating the albumin quotient.

Further complicating the interpretation is the fact that the presence of intrathecally produced immunoglobulins can also elevate CSF protein levels. Unfortunately, routine checks for intrathecal immunoglobulin synthesis, crucial for differential diagnosis, are often bypassed. Thus, intrathecally produced immunoglobulins can result in misleadingly elevated total CSF protein readings.

Moreover, the relationship between age and both the Q_Alb_ and total CSF protein is another point of contention. Studies by Castellazzi et al. (22) and McCudden et al. (23) indicate a positive correlation between these parameters and age. Yet, in clinical practice, the interpretation of total CSF protein is often done without considering age, given the standard, non-age-dependent cut-off values.

Using the fixed cut-off (usually 500 mg/L) value for total protein is particularly dangerous in potentially misdiagnosing blood-CSF-barrier dysfunction among younger individuals. In such cases, if the total protein is below this threshold, it might yield false negative outcomes. Conversely, elderly patients with a well-functioning blood-CSF-barrier might show a falsely elevated total CSF protein level, leading to false positive results.

In patients with immune-mediated neuropathies, the presence of a blood-liquor barrier dysfunction is important for assessing whether, for example, CIDP is imminent.

CIDP represents a widely known inflammatory neuropathy typically involving symmetrical, sensorimotor neuronal damage and - in contrast to other rapidly progressing entities like the Guillain Barré Syndrome - manifesting with a slowly progressive onset but often leading to severe disability and consecutive reduction in quality of life (24–27). As evidence a blood-liquor barrier dysfunction represents one of the key diagnostic features, a reliable measurement is essential in prompting the correct diagnosis and implementing adequate treatment administration (28).

To investigate the applicability of both Q_Alb_ and total CSF protein in this particular context, this multicenter analysis in patients with immune neuropathies was conducted. In our multicenter study encompassing 419 samples, we observed a significant divergence between the CSF total protein and Q_Alb_ in 8.6% of cases involving patients with immune-mediated neuropathies. In particular, 2.3% of the patients showed a normal total protein value, although the albumin quotient was elevated and thus showed a blood-CSF barrier dysfunction. As hypothesized, these were especially younger patients in whom a blood-CSF barrier dysfunction would otherwise have been mistakenly overlooked if only the total protein had been examined. To our knowledge, this represents a relevant a novel finding that has been hinted at in only one smaller cohort of 110 patients with GBS (29).

We were also able to confirm the correlation of Q_Alb_ and total CSF protein values as previously established (29). Additionally, a marginal inverse correlation of Q_Alb_ and MRC Sum Score was also evident in our cohort. This finding most likely expresses the quantitative interrelation of blood-CSF-barrier dysfunction and severity of disease progression of immune-mediated neuropathies. Interestingly, this correlation has not yet been thoroughly investigated and was only negative in patients with amyotrophic lateral sclerosis (30).

An additional finding of our analysis was that samples derived from male patients showed significantly higher values for Q_Alb_ and total CSF protein. This finding is in line with previously published studies showing the same effect in different cohorts of neurological and psychiatric background (22, 31, 32).

The consecutive separate subgroup analyses for male and female patients, respectively, showed that especially samples from female patients derived from significantly younger patients at onset, diagnosis and lumbar puncture when comparing those with isolated elevated Q_Alb_ (and normal total CSF protein) versus samples with elevated total CSF protein (independent of Q_Alb_ values). We therefore deduced that especially female patients of younger age at onset, diagnosis and at lumbar puncture were prone to be underdiagnosed in terms of blood-CSF-barrier-dysfunction when neglecting Q_Alb_ as the primary biomarker and focusing on the hitherto established sole total CSF protein levels instead.

The main strengths of this manuscript are the relevant cohort size and the approach in a multicenter manner. However, data interpretation is limited by the retrospective study design and the non-standardized CSF collection.

## 5 Conclusion

In conclusion, our research supports the known risks of blood-CSF-barrier-evaluation depending solely on total protein measurements, especially in younger cohorts. The pivotal importance of Q_Alb_ in diagnostics is evident. Excessive dependence on total protein can mislead interpretations, risking precise and prompt clinical decisions. Our findings strongly support for incorporating Q_Alb_ evaluations, crucial for achieving reliable results, particularly in younger patients with immune-mediated neuropathies.

## Data availability statement

The raw data supporting the conclusions of this article will be made available by the authors, without undue reservation.

## Ethics statement

The studies involving humans were approved by local ethics committees MHH: 8172_BO_K_2018, Ruhr-University Bochum: 18-6534-BR, Cologne: 21–1,079, Berlin: EA4/166/23. The studies were conducted in accordance with the local legislation and institutional requirements. The participants provided their written informed consent to participate in this study.

## Author contributions

TSe: Conceptualization, Data curation, Formal analysis, Writing – original draft. SG: Data curation, Writing – review & editing. YG: Data curation, Writing – review & editing. LH: Data curation, Writing – review & editing. BL: Data curation, Writing – review & editing. FK: Data curation, Methodology, Writing – review & editing. HP: Data curation, Writing – review & editing. FS: Data curation, Methodology, Writing – review & editing. JM: Data curation, Writing – review & editing. AF: Data curation, Writing – review & editing. TG: Data curation, Writing – review & editing. KP: Supervision, Writing – review & editing. TSk: Conceptualization, Supervision, Writing – review & editing.
