# Supplementary material for: Comparative analysis of albumin quotient and total CSF protein in immune-mediated neuropathies: a multicenter study on diagnostic implications
Source: Front Neurol. 2024 Jan 9;14:1330484. doi: 10.3389/fneur.2023.1330484 (PMC10803547; doi:10.3389/fneur.2023.1330484)
Supplement: Supplementary file 1 [file Table_1.docx]

Supplementary table 1. Detailed logistic regression analysis on samples with elevated Q-Albumin and samples with elevated total CSF protein values for sex-, age- and disability-dependent effects

| Analysis for all samples with elevated Q-Alb (N=246 [58%]) | | | | |
| --- | --- | --- | --- | --- |
| Variable | Univariate OR [95%CI] | Univariate p-value | Multivariate OR [95%CI] | Multivariate p-value |
| Female sex | 0.42 [0.27-0.64] | **<0.001** | 0.39 [0.26-0.61] | **<0.001** |
| Age at onset | 9.88 [0.97-1.002] | 0.1 | n/i | n/i |
| Age at diagnosis | 0.96 [0.97-1.001] | 0.06 | n/i | n/i |
| Age at lumbar puncture | 0.98 [0.97-0.999] | **0.04** | 0.98 [0.97-0.996] | **0.01** |
| INCAT at time of lumbar puncture | 1.09 [0.98-1.2] | 0.1 | n/i | n/i |
| MRC Sum Score at time of lumbar puncture | 0.99 [0.97-1.01] | 0.1 | n/i | n/i |
| Diagnosis | 0.97 [0.92-1.03] | 0.3 | n/i | n/i |
| Analysis for all samples with elevated total CSF protein values | | | | |
| Variable | Univariate OR [95%CI] | Univariate p-value | Multivariate OR [95%CI] | Multivariate p-value |
| Female sex | 0.46 [0.3-0.7] | **<0.001** | n/i | n/i |
| Age at onset | 1.0 [0.99-1.01] | 0.96 | n/i | n/i |
| Age at diagnosis | 1.0 [0.98-1.01] | 0.95 | n/i | n/i |
| Age at lumbar puncture | 1.0 [0.98-1.01] | 0.87 | n/i | n/i |
| INCAT at time of lumbar puncture | 1.1 [0.995-1.23] | 0.06 | n/i | n/i |
| MRC Sum Score at time of lumbar puncture | 0.98 [0.96-1.003] | 0.08 | n/i | n/i |
| Diagnosis | 0.97 [0.92-1.03] | 0.3 | n/i | n/i |

Q-Alb - Q-Albumin, N - number, OR - Odds Ratio, n/i - not included into multivariate regression model, CI - confidence interval
