# Supplementary figures and images for: Comparative analysis of albumin quotient and total CSF protein in immune-mediated neuropathies: a multicenter study on diagnostic implications
Source: Front Neurol. 2024 Jan 9;14:1330484. doi: 10.3389/fneur.2023.1330484 (PMC10803547; doi:10.3389/fneur.2023.1330484)

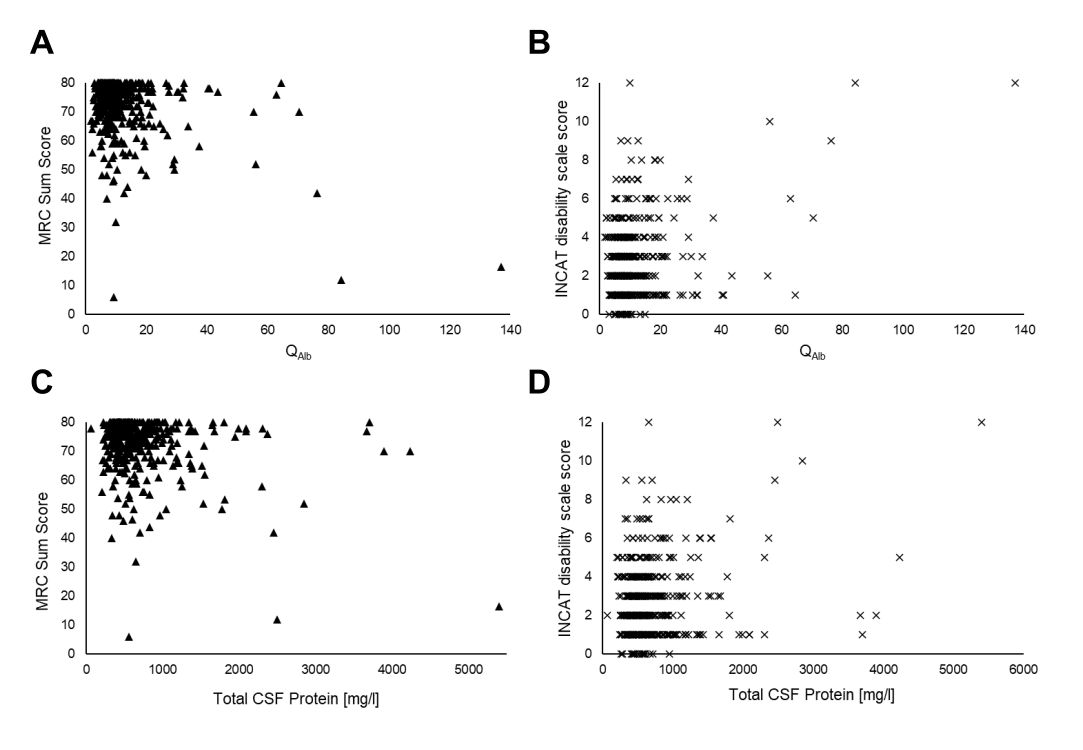

Supplement: Supplementary file 2 [file Image_1.TIF]
